# Regulation of Necroptosis by Phospholipids and Sphingolipids

**DOI:** 10.3390/cells9030627

**Published:** 2020-03-05

**Authors:** Xuewei Zhang, Masaya Matsuda, Nobuo Yaegashi, Takeshi Nabe, Kazuyuki Kitatani

**Affiliations:** 1 Department of Obstetrics and Gynecology, Tohoku University Graduate School of Medicine, Tohoku University, Sendai 980-8574, Japan; zhangxuejian9521@yahoo.co.jp (X.Z.); yaegashi@med.tohoku.ac.jp (N.Y.); 2 Laboratory of Immunopharmacology, Faculty of Pharmaceutical Sciences, Setsunan University, Hirakata 573-0101, Japan; masaya.matsuda@pharm.setsunan.ac.jp (M.M.); t-nabe@pharm.setsunan.ac.jp (T.N.)

**Keywords:** necroptosis, lipids, ceramide, fatty acids, phosphatidylinositols, mixed lineage kinase-domain like

## Abstract

Several non-apoptotic regulated cell death pathways have been recently reported. Necroptosis, a form of necrotic-regulated cell death, is characterized by the involvement of receptor-interacting protein kinases and/or the pore-forming mixed lineage kinase domain-like protein. Recent evidence suggests a key role for lipidic molecules in the regulation of necroptosis. The purpose of this mini-review is to outline the regulation of necroptosis by sphingolipids and phospholipids.

## 1. Introduction

Cell death can be generally divided into two types: apoptosis and necrosis. Apoptosis is characterized by activation of caspases and typical morphological features such as nuclear condensation, cell shrinkage, and membrane blebbing [1,2]. For decades, apoptosis was described as the only form of regulated cell death. In contrast, necrosis was recognized as a form of uncontrolled passive cell death with classical necrotic morphologies such as cytoplasmic membrane breakdown and cellular organelle swelling [3]. However, accumulating evidence has recently revealed the existence of multiple forms of necrotic-regulated cell death such as necroptosis, autosis, ferroptosis, NETosis, pyroptosis, and several others [4,5]. A number of studies have demonstrated important links between these cell death pathways and organismal homeostasis, as well as several infectious, pulmonary, cardiovascular, neurodegenerative, and hepatic diseases, and cancer [6,7,8]. Therefore, understanding the regulation of necrotic-regulated cell death pathways is of great biomedical interest.

Various classes of lipids in eukaryotic cells have been identified as bioactive signaling molecules in regulated cell death [9,10,11,12,13]. Accumulating evidence suggests crucial roles for glycerophospholipids, sphingolipids, and fatty acids in necroptosis.

### 1.1. Necroptosis

Necroptosis is a form of regulated cell death with hallmark features of necrosis. At the molecular level, necroptosis critically depends on the sequential activation of receptor-interacting protein kinase 1 (RIPK1), RIPK3, and the pore-forming mixed lineage kinase domain-like protein (MLKL) independently of caspases [4,5,6,8]; the interaction of MLKL with lipids is needed for the execution of necroptosis [14,15,16]. Necroptosis induction has been shown to occur in response to oxidative stress, DNA damage, and reagents including tumor necrosis factor α (TNF-α), ceramide nanoliposomes, chemotherapeutic reagents, and fingolimod [4,5,8,17,18,19,20]. The signal transduction pathway of necroptosis involves a signaling complex, called the necrosome, which contains RIPKs and MLKL. The molecular mechanisms underlying TNF-α-induced necroptosis are the most well-defined [21,22,23]. Briefly, upon stimulation of the death receptor TNF receptor 1 by TNF, a signaling complex containing TRADD, TRAF2/5, LUBAC, RIPK1, and cIAP1/2 is formed, termed Complex I [24]. Complex I initiates nuclear factor κB activation and mitogen-activated protein kinase pathways. The formation of Complex II is trigged by non-ubiquitinated RIPK1. When caspase 8 is inactive and high levels of RIPK3 exist, RIPK3 is recruited by RIPK1, leading to formation of the necrosome.

Activated RIPK3 phosphorylates the pore-forming protein MLKL, which facilitates the oligomerization of MLKL and its subsequent translocation to the plasma membrane [25]. Recent studies suggest that MLKL oligomers form cation channels on the plasma membrane, leading to high osmotic pressure, water influx, release of intracellular components, and eventual plasma membrane rupture [14,26,27,28,29]. In short, targeting MLKL to the plasma membrane is an executional step during necroptosis.

### 1.2. MLKL

**Figure 1:**
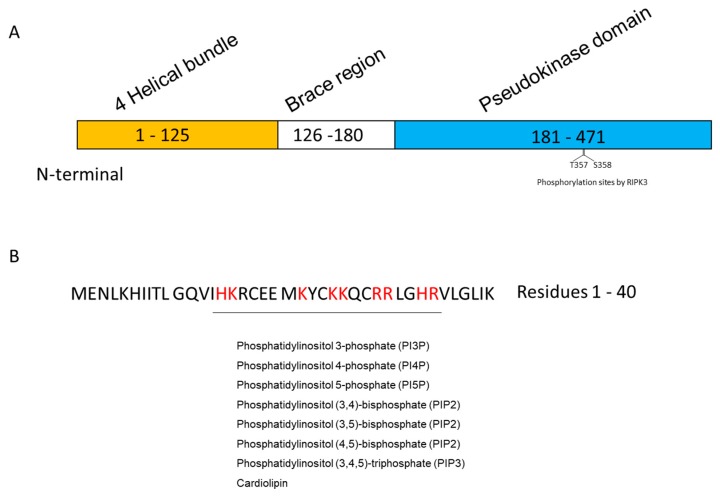
MLKL structure and phospholipid-binding region. (**A**) Domains of human MLKL; (**B**) Amino acids (red) responsible for binding to phospholipids. MLKL, mixed lineage kinase domain-like protein.

MLKL consists of an N-terminal four-helical bundle domain (1–125 amino acids (a.a.)), a brace domain (126–180 a.a.), and a C-terminal kinase-like domain (181–471 a.a.) [14]. The N-terminal region contains membrane-interacting regions with negative charged phospholipids. In contrast, the C-terminal kinase-like domain contains RIPK3 phosphorylation sites (T357 and S358) (Figure 1A). These domains are suggested to modulate MLKL oligomerization and membrane targeting for necroptosis execution.

MLKL oligomerization is a remarkable feature of necroptotic cell death. The translocation of oligomerized MLKL to the cell plasma membrane is a critical step during necroptosis. In 2014, Cai et al. discovered that MLKL oligomerization induces Ca^2+^ influx mediated by transient receptor potential melastatin-related 7, eventually causing cell death [26]. Further studies using liposomes demonstrated that MLKL oligomerization caused membrane leakage [14,27]. These results suggest that MLKL oligomers form a pore in the plasma membrane to cause the release of cellular contents, ionic homeostasis, and cell rupture. Moreover, Xia et al. proposed that MLKL forms cation channels [28].

Considering the unique role of MLKL in necroptosis, the biological relevance of MLKL in specific organelles has also been studied. Recently, Liu et al. showed that lysosomal damage leads to accumulated MLKL expression after spinal cord injury, which sensitizes cells to necroptosis [30]. Data from the Vandenabeele laboratory indicated that RIPK3-induced MLKL phosphorylation occurs in the nucleus, suggesting that MLKL is a nucleocytoplasmic shuttling protein [31]. Moreover, Wang et al. reported in 2014 that phospho-MLKL translocated to mitochondrial membranes following necroptosis induction of human colon cancer cells. These findings, which clarify the molecular mechanisms involved in MLKL-mediated necroptosis, suggest that MLKL is the most important molecule that eventually executes cell death during necroptosis.

### 1.3. Lipids

Lipids are recognized as permeability barriers of cells. However, in addition to their classical roles in energy storage or as structural molecules, lipids have emerged as essential signaling regulators of inflammatory responses, cell proliferation, differentiation, motility, and death [10,32,33,34,35,36,37,38,39,40,41]. Given the dramatic changes that occur in cell membranes during necroptosis, several research groups have considered lipids to participate and have important roles in this process. Recently, emerging evidence has suggested the significance of lipidic molecules (fatty acids [42], sphingolipids [20,43,44], and glycerophospholipids [14,15]) in the regulation of necroptotic cell death. In addition, lipid rafts have been proposed to play an essential role in the necroptotic cell death pathway [25].

Novel functions of lipids in the execution of necroptosis have increasingly attracted researchers’ attention. In this review, we summarize the functional involvement of phospholipids and sphingolipids in necroptosis.

## 2. Phospholipids

The functions of plasma membrane-associated proteins are often controlled by a number of protein domains (such as C1, C2, and PH) that recognize specific lipid molecules in membranes [45]. Negatively charged membrane phospholipids are believed to electrostatically interact with positively charged amino acids in a domain of the membrane-binding protein.

Multiple research groups have demonstrated that MLKL interacts with negatively charged phospholipids including phosphorylated phosphatidylinositols and cardiolipins [14,15,27]. These phospholipids efficiently bind to MLKL and the interaction is believed to play an important role in recruitment of MLKL to the membrane.

### 2.1. Inositol Phospholipids

MLKL contains a region that is rich in positively charged amino acids (His15, Lys16, Lys22, Lys25, Lys26, Arg29, Arg30, His33, and Arg34) in four helical bundles. Dondelinger et al. [14] demonstrated that these positively charged amino acids are required for MLKL binding to phosphorylated phosphatidylinositols and cardiolipins, as well as its recruitment to the plasma membrane (Figure 1B). Importantly, ectopic expression of MLKL caused cell death, whereas an MLKL mutant with replacement of all nine positively charged amino acids with negatively charged amino acids abolished programmed cell death. These results implicate the interaction of MLKL with negatively charged phospholipids in the regulation of necroptosis.

Recently, it was reported that binding of a highly phosphorylated inositol phosphate promoted MLKL to adopt an active conformation by displacing the N-terminal auto-inhibitory brace, thereby raising the possibility that inositol phosphate kinase activity is a crucial requirement for MLKL necroptotic function [46]. Data from the Green laboratory proposed a stepwise activation mode of MLKL during necroptosis [15]. The MLKL brace region, which is close to the N-terminal helix bundle, is responsible for MLKL oligomeric conformation and recruitment to the plasma membrane. In this process, MLKL is targeted to the plasma membrane via low-affinity binding of Lys16/Arg17 in the α1 helix of the N-terminal bundle to the polar head groups of phosphatidylinositol(4,5)-bisphosphate. Subsequently, the N-terminal helix bundle goes through a ‘rolling over’ mechanism to expose higher-affinity phosphatidylinositol(4,5)-bisphosphate-binding sites (α2 helix of the N-terminal bundle) responsible for strong binding to the plasma membrane. Thus, phosphatidylinositol(4,5)-bisphosphate is a key lipid mediator in the necroptosis signaling pathway.

Furthermore, in a cell-free assay, MLKL was shown to induce leakage of phosphatidylinositol phosphate-containing liposomes, thereby proposing a model in which MLKL induces necroptosis by permeabilizing the plasma membrane [14].

### 2.2. Cardiolipin

Cardiolipin is a mitochondrial-specific lipid that binds to the N-terminal four-helical bundle domain of MLKL [27]. The cardiolipin-MLKL interaction was suggested to potentiate MLKL-dependent permeabilization of membranes [27]. Moreover, phosphorylated MLKL colocalized with mitochondria, endoplasmic reticulum, lysosomes, and plasma membranes of HT-29 cells. In light of the observed mitochondrial localization, phosphorylated MLKL may also permeabilize cardiolipin-enriched mitochondrial membranes. However, biological significance of phosphorylated MLKL localization to endoplasmic reticulum and lysosomes remains fully unknown.

Wang et al. demonstrated that phosphorylated MLKL transduced the necroptotic signal by forming protein complexes with RIPK1, RIPK3, and mitochondrial phosphatase PGAM5 [47]. PGAM5 was shown to dephosphorylate and activate the mitochondrial fission protein Drp1, and the consequent Drp1-mediated fragmentation of mitochondria appeared to be an obligatory step for necrosis execution. However, recent studies showed that mitochondria-depleted cells still died of necroptosis [48,49]. Thus, further studies are required to understand the biological significance of mitochondrial MLKL in necroptosis.

## 3. Sphingolipids

Characterized by their backbone of sphingoid bases (sphingosine and dihydrosphingosine), sphingolipids are components of eukaryotic cells, many of which function as bioactive signaling molecules. Ceramide, a central molecule in the sphingolipid metabolic pathway, plays a vital role in mediating cell death [12,37]. In terms of cancer biology, ceramide has been proposed to function as an anti-tumor and anti-metastatic lipid, thereby suggesting the potential of ceramide-based therapy for cancer. Recent studies of ceramide-based cancer therapies revealed that treatment of ovarian cancer cells with ceramide nanoliposomes [50] evoked MLKL-driven necroptosis independently of RIPK1/3 [20]. In this section, we outline the role of ceramide-centered sphingolipids as regulatory mediators of necroptosis.

### 3.1. Ceramide

Ceramide serves as an apoptotic lipid. Recently, several groups have implicated ceramide in necroptosis induction. In 2014, Wang and colleagues showed for the first time that short-chain ceramide (C_2_-ceramide) induced programmed necrosis [51]; however, the molecular mechanism has yet to be elucidated. Ogretmen’s group proposed that ceramide and RIPK1 form a complex that assembles a large membrane pore (named ceramidosome), which is responsible for blebbing and necroptosis [44]. Zhang et al. discovered that liposomal ceramides facilitated MLKL oligomerization responsible for necroptosis [20].

**Figure 2:**
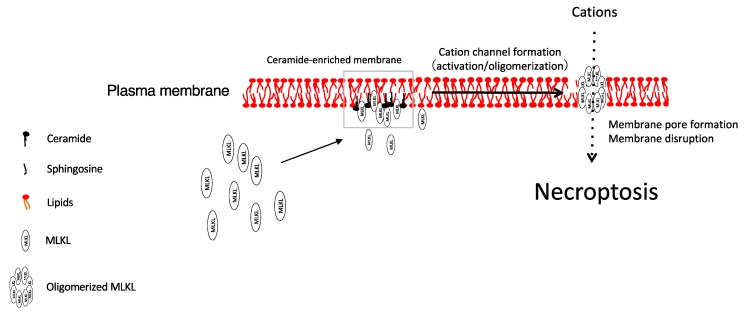
Proposed model for necroptosis induced by ceramide.

Various nanoscale formulations for ceramide have been developed to treat cancer [50]. Our research group found an antitumor effect of nanoliposomal ceramides in ovarian cancer and proposed a novel mechanism of action [20,52]. Our data [20] indicated that treatment of ovarian cancer cells with ceramide nanoliposomes decreased cell viability in a dose- and time-dependent manner. Importantly, ceramide nanoliposome-treated ovarian cancer cells died by necrosis, but not apoptosis. Mechanistically, dying ovarian cancer cells exhibit oligomerization and relocalization to plasma membrane of MLKL, which is a hallmark of necroptosis. Moreover, knockdown of MLKL, but not its upstream molecules RIPK1/3, significantly abolished ceramide nanoliposome-induced cell death. These results suggest that ceramide nanoliposomes target MLKL, leading to necroptosis in ovarian cancer cells (Figure 2). Although the molecular mechanism of ceramide nanoliposome-induced MLKL activation is still unknown, it is assumed that ceramide and/or its metabolites function as regulatory molecules in the necroptosis signaling pathway.

In addition, our data demonstrated that systemic administration of ceramide nanoliposomes suppressed metastatic growth in an ovarian cancer xenograft model. Taken together, these findings suggest that ceramide nanoliposomes elicit a cytotoxic effect by inducing MLKL-dependent necroptosis in ovarian cancers. Thus, ceramide nanoliposomes are suggested to serve as potential necroptosis-inducing, chemotherapeutic reagents for ovarian cancer.

Accumulation of endogenous ceramide was shown to promote necroptotic cell death in human luteal granulosa cells, implicating the possible involvement of ceramide synthases in necroptosis induction [53]. More recent studies indicated that upregulation of C_16_-ceramide triggered mitophagy-mediated necroptosis, suggesting important crosstalk between ceramide production and necroptotic cell death in lung structural cells [54]. Moreover, an independent research study demonstrated that excessive C_16_-ceramide content under conditions of caspase inactivation promoted necroptosis in trophoblast cells [55]. Collectively, these findings suggest that ceramide may serve as a necroptosis-inducing lipid.

### 3.2. Sphingosine Analogue FTY720 (Fingolimod)

FTY720, an artificially synthesized sphingosine analogue is thought to function as a first-in-class sphingosine 1-phosphate receptor modulator. Recently, a novel role for FTY720 in necroptosis was reported, as it was shown to directly target inhibitor 2 protein phosphatase 2A (also known as SET) via a lipid–protein interaction, leading to protein phosphatase 2A (PP2A) activation and subsequent induction of RIPK1-dependent necroptosis [17]. However, whether FTY720-induced PP2A activation directly regulates RIPK1 for the induction of necroptosis remains unknown. Moreover, recent advances suggest that FTY720 promotes the formation of C_16_-ceramide- and RIPK1-enriched membrane pores, referred to as ceramidosomes [44], which likely regulate membrane integrity and signaling in necroptosis.

## 4. Very Long Chain Fatty Acids

Parisi et al. demonstrated that very long chain fatty acids play a functional role in necroptosis [42]. During necroptosis, ceramides and very long chain fatty acids accumulate in a RIPK1-dependent manner. Biochemically, activated fatty acid biosynthesis and elongation likely contribute to this accumulation. Importantly, suppression of the formation of very long chain fatty acids inhibited plasma membrane rupture. Thus, very long chain fatty acids are possibly involved in the loss of plasma membrane integrity in necroptosis, as these findings suggest that they may correlate with membrane permeabilization in response to necroptosis induction. As such, identification of these lipid-associated targets will be a task for future studies.

## 5. Glycolipid Transfer Protein (GLTP)

In addition to lipidic molecules, several lipid-associated proteins have been reported as potential regulators during necroptosis. GLTP is known to mediate intermembrane trafficking of glycosphingolipids and regulate their homeostatic levels within cells [56,57]. Mechanistically, upregulation of human GLTP was shown to promote RIPK3-dependent MLKL activation, which increased expression levels of Kip1/p27 and Cip1/p21, and induced Ca^2+^ release from internal stores to trigger necroptosis in colon carcinoma cells [58].

## 6. Docosahexaenoic Acid (DHA)

A study performed in 2014 shed light on involvement of the omega-3 fatty acid DHA in TNF-α-induced necroptosis [59]. DHA inhibited TNF-α-induced necroptosis and decreased long-chain C_16_-C_18_ ceramides. These findings suggest that DHA may suppress TNF-α-mediated necroptosis by interfering with sphingolipid metabolism. However, the underlying molecular mechanisms need to be investigated.

## 7. Future Outlook and Concluding Remarks

Necroptosis is recognized as a non-apoptotic form of regulated cell death that is dependent on RIPK1/3 and/or MLKL protein activation. Rigorous studies suggest that specific lipids function as key protein-regulating molecules during necroptosis. However, future studies regarding lipid–protein interactions are needed to elucidate the lipid-driven signaling pathway of necroptosis.
